# Assessing severe acute respiratory coronavirus virus 2 (SARS-CoV-2) preparedness in US community hospitals: A forgotten entity

**DOI:** 10.1017/ice.2020.1238

**Published:** 2020-10-07

**Authors:** Sonali D. Advani, Esther Baker, Andrea Cromer, Brittain Wood, Kathryn L. Crawford, Linda Crane, Linda Adcock, Linda Roach, Polly Padgette, Deverick J. Anderson, Daniel J. Sexton

**Affiliations:** 1 Duke Center for Antimicrobial Stewardship and Infection Prevention, Durham, North Carolina; 2 Duke University School of Medicine, Department of Internal Medicine, Durham, North Carolina

## Abstract

We performed a cross-sectional survey of infection preventionists in 60 US community hospitals between April 22 and May 8, 2020. Several differences in hospital preparedness for SARS-CoV-2 emerged with respect to personal protective equipment conservation strategies, protocols related to testing, universal masking, and restarting elective procedures.

Novel coronavirus (SARS-CoV-2) has been associated with the largest recorded coronavirus outbreak to date. In the United States, there have been >2.2 million cases with >119,000 deaths (as of June 21, 2020).^1^ This pandemic has placed a tremendous strain on the US healthcare system leading to personal protective equipment (PPE) and resource shortages.^2^ Most hospitals have implemented contingency and crisis capacity strategies to optimize the use of resources.^3^

Although public health agencies like the Centers for Disease Control and Prevention (CDC) have provided interim guidance on infection prevention and control in US hospitals,^4^ the current state of community hospital preparedness is unknown. Assessing preparedness of community hospitals is crucial to risk assessments and outbreak control activities in these settings. Hence, we conducted a cross-sectional survey of SARS-CoV-2 preparedness among community hospitals in southeastern United States.

## Methods

### Survey design and setting

We performed a cross-sectional survey of 60 community hospitals within the Duke Infection Control Outreach Network (DICON). DICON provides infection control services to 60 community hospitals and surgery centers in 6 states (North Carolina, South Carolina, Virginia, Florida, Georgia, and West Virginia).^5^ These hospitals range in size from 30 to 685 beds, with a median size of 162 beds. Also, 77% of these hospitals have maternity and pediatric wards. This study was deemed exempt from institutional review board review by the Duke University Health System (no. Pro00105818).

### Survey instrument and distribution

The survey (provided in the Supplementary Data online) was conducted between April 22 and May 5, 2020, using Qualtrics (Qualtrics, Provo, UT); it was distributed electronically to infection preventionists at community hospitals. Participation was voluntary, anonymous, and without compensation. The survey included 13 questions related to PPE availability, crisis capacity strategies to extend and reuse PPE, policies related to restarting surgeries, testing prior to elective surgery and prior to transfer to extended care facilities, universal masking, and daily screening of hospital staff. Extended use was defined as using the same single-use PPE for encounters with multiple patients without removing it between encounters. Reuse was defined as using the same PPE for multiple encounters but doffing it after each encounter and donning it prior to the next encounter. Survey responses were analyzed using descriptive statistics.

## Results

**Fig. 1.**
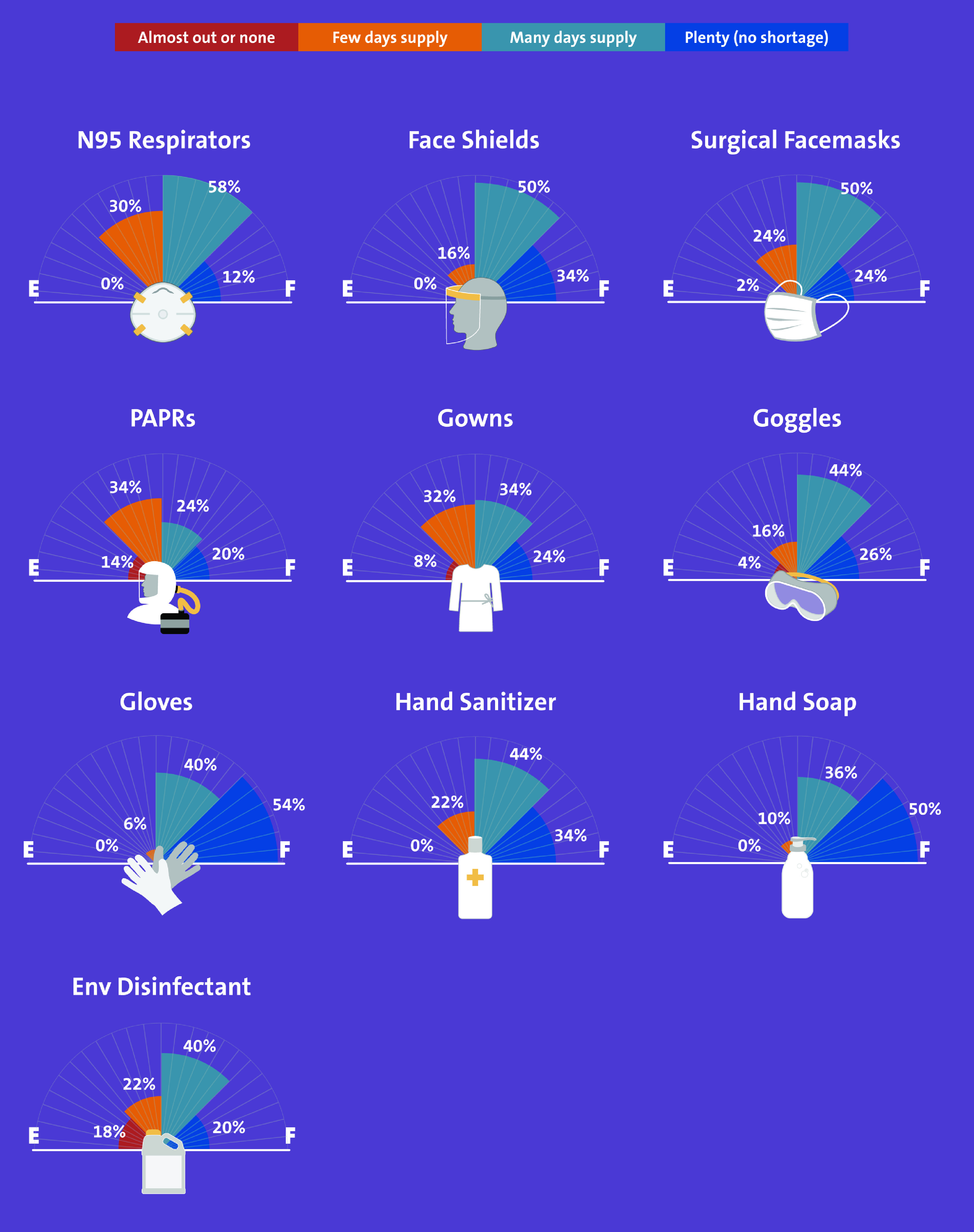
Supply of resources in 50 community hospitals in the southeastern United States.

Of 60 hospitals, 50 (83%) responded to our survey. These hospitals reported varying degrees of PPE shortages (Fig. 1). Overall, 20 hospitals (40%) reported “no supply” or “few days supply” of powered air-purifying respirators (PAPRs), environmental disinfectant, and gowns. Almost 30% of facilities reported an insufficient supply of face masks and N95 respirators, and 16% reported an insufficient supply of face shields. More than 80% of community hospitals were implementing strategies to reuse N95 respirators, face shields, and goggles. Only 6 hospitals (12%) were reusing gowns at the time of this survey. Similarly, at least 80% of hospitals were extending the use of N95 respirators, face shields, and surgical masks (Table 1). Furthermore, 36 community hospitals (72%) reported reprocessing N95 respirators, mostly using hydrogen peroxide plasma (29.8%), ultraviolet radiation (21%), and/or hydrogen peroxide vapor (12%).

**Table 1.** Distribution of Community Hospitals That Extend the Use of Their Personal Protective Equipment (PPE) or Reuse Their PPE

Type of PPE	Reuses PPE, No. (%)	Extends PPE, No. (%)
N95 respirators	43 (86)	44 (88)
Face shields	46 (92)	40 (80)
Surgical facemasks	32 (64)	38 (76)
Gowns	6 (12)	10 (20)
Gloves	0 (0)	2 (4)
Goggles	41 (82)	30 (60)
PAPRs	38 (76)	25 (50)

Note. PAPR, powered air-purifying respirator.

Most community hospitals had implemented universal masking policies: 38 (76%) required masking of patients, visitors and healthcare personnel (HCPs), 7 (14%) required masking of HCPs and visitors; and 4 (8%) required universal masking of HCPs only. Also, 90% of hospitals were performing daily employee screening at point of entry. Additionally, 7 (14%) hospitals had restarted tier 1 elective surgical procedures at the time of this survey; 16 (32%) restarted tier 2 nonurgent surgical procedures; and 43 (86%) were performing only tier 3 emergent surgical procedures. Only 43 facilities (86%) reported performing preoperative testing for SARS-CoV-2. Moreover, 17 facilities (34%) performed 1 SARS-CoV-2 PCR test before discharging an asymptomatic patient to skilled nursing facilities, and 20 facilities (40%) performed 2 tests prior to discharge to these facilities. The community hospitals in our network reported a wide variety of laboratories used for SARS-COV-2 testing, with most using in-house testing (n = 34, 68%), followed by testing by LabCorp (n = 21, 42%), Quest Diagnostics (n = 13, 42%), Department of Health (n = 13, 26%), and others. Only 4% of hospitals performed antibody testing for SARS-COV-2 at the time of this survey.

## Discussion

The results of this survey reveal gaps and differences in SARS-COV-2 preparedness among community hospitals in the southeastern United States. A recent survey of hospitals in the Society for Healthcare Epidemiology of America Research Network highlighted similar shortages in academic hospitals and large medical centers,^6^ but our survey is the first report, to our knowledge, focusing on the state of smaller community hospitals during the COVID-19 pandemic.

Almost half of the community hospitals reported shortages in their supplies of PAPRs, environmental disinfectants, and gowns. In addition, 80% of hospitals reported an adequate supply of N95 respirators, face shields, and googles, likely due to use of crisis capacity strategies to extend, reuse, and reprocess these PPE. Our report is different from a recently reported survey of hospitals in Idaho that reported shortages of face shields.^7^ Our survey highlights that face shields are less prone to shortages due to their simpler design, reuse potential, and durability.^8^

More than half of the community hospitals in our network had employed strategies to extend the use of face masks, N95 respirators, gowns, face shields, and googles. Currently, to our knowledge, no data are available on the safety of extended use PPE or time limits for safely extending the use. Similarly, most hospitals were employing strategies to reuse N95 respirators, PAPRs, face shields, goggles, and masks. Although some data exist on the safety of reprocessed N95 respirators, safety data on reuse of other single use PPE are scarce.^9^ Shortages of disinfectants and sanitizers may lead to the introduction of new agents with a potential decrease in cleaning efficiency, variation in equipment compatibility, an increase in staff dissatisfaction, and occupational safety hazards.^9^

Our survey also demonstrates that most of our community hospitals had implemented policies related to employee screening at the point of hospital entry. Although most hospitals had developed policies related to universal masking, the content of these policies varied widely. There was significant variation in policies related to testing for active infection with SARS-CoV-2 infection, with respect to the laboratory used, testing before surgical procedures, and testing prior to discharge to skilled nursing facilities.

Our study has several limitations. It was a cross-sectional study and relied on self-reported data from infection preventionists. We did not include other healthcare facilities such as nursing homes. However, this survey provided valuable information on differences in outbreak readiness among community hospitals that may help identify factors influencing preparedness.

We found several differences in community hospital preparedness for SARS-CoV-2 with respect to type of conservation strategies used to preserve PPE, protocols related to testing, masking, and restarting elective procedures. We believe that this lack of standardization in approaches was due to differences in state guidelines, the decentralized federal approach to SARS-CoV-2 preparedness, and a lack of confidence in public health guidelines. These differences also highlight the challenges with implementing guidelines related to SARS-CoV-2 in community hospitals because of PPE and personnel shortages, financial constraints, and uncertainty regarding how and when to implement policies such as universal masking, preoperative testing, and predischarge testing. This study also offers a starting point for future assessments of pandemic preparedness among community hospitals in the United States.

## Supplementary material

For supplementary material accompanying this paper visit https://doi.org/10.1017/ice.2020.1238. click here to view supplementary material
