# Supplementary material for: Assessing severe acute respiratory coronavirus virus 2 (SARS-CoV-2) preparedness in US community hospitals: A forgotten entity
Source: Infect Control Hosp Epidemiol. 2020 Oct 7:1–4. doi: 10.1017/ice.2020.1238 (PMC7582016; doi:10.1017/ice.2020.1238)
Supplement: Supplementary file 1 [file S0899823X20012386sup001.pdf]

DICON SARS-COV-2 Preparedness Survey Projects Contacts Actions Library Help

Survey Actions Distributions Data & Analysis Reports

DICON SARS-COV-2 Preparedness Survey

iQ Score: Great Published

Default Question Block Block Options

Q2

Name of facility:

Q2

Does this facility have adequate supply of the following?

	Plenty (No shortage anticipated)	Many days supply	Few days supply	Almost out or none	Unknown
N95 respirators	◯	◯	◯	◯	◯
Face shields	◯	◯	◯	◯	◯
Surgical facemasks	◯	◯	◯	◯	◯
PAPRs	◯	◯	◯	◯	◯
Gowns	◯	◯	◯	◯	◯
Goggles	◯	◯	◯	◯	◯
Gloves	◯	◯	◯	◯	◯
Hand Sanitizer	◯	◯	◯	◯	◯
Hand Soap	◯	◯	◯	◯	◯
Environmental cleaner/disinfectant	◯	◯	◯	◯	◯

Q3

Does this facility EXTEND the use of the following (i.e. use same N95 for repeated close encounters with different patients w/o removing with exception of AGPs) ?

	Yes	No	Unknown
N95 respirators	◯	◯	◯
Face shields	◯	◯	◯
Surgical facemasks	◯	◯	◯
Gowns	◯	◯	◯
Gloves	◯	◯	◯
Goggles	◯	◯	◯
PAPR	◯	◯	◯

Q4

Does this facility REUSE any of the following (i.e., same N95 for multiple encounters but removing between encounters)?

	Yes	No	× Unknown
N95 respirators	○	○	○
Face shields	○	○	○
Surgical facemasks	○	○	○
Gowns	○	○	○
Gloves	○	○	○
Goggles	○	○	○
PAPR	○	○	○

Q5

Is this facility reprocessing N95 respirators using the following methods?

- ☐ Hydrogen Peroxide Vapor (Bioquell etc)
- ☐ Hydrogen Peroxide Vapor (STERIS V-PRO)
- ☐ Hydrogen Peroxide Plasma (STERRAD)
- ☐ UV radiation
- ☐ Ethylene oxide
- ☐ Other, enter method below:
- ☐ Planning to reprocess using method chosen, not started yet
- ☐ Does not reprocess N95

Q6

Is this facility performing any of the following surgeries/procedures?

- ☐ Tier 1 Elective procedures (Carpal tunnel release, EGD, Colonoscopy, Cataracts, etc)
- ☐ Tier 2 Nonurgent procedures (Low risk cancer, Non-urgent spine & Ortho: Including hip, knee replacement and elective spine surgery, Stable ureteral colic, Elective angioplasty)
- ☐ Tier 3 Emergent (Most cancers, Neurosurgery, Highly symptomatic patients, cardiac w symptoms, trauma, vascular surgery, limb threatening etc)
- ☐ Other: Enter comment below

Q7

Is this facility testing or required to test asymptomatic patients before transfer to long term care or skilled nursing facilities?

- ○ Yes, one test
- ○ Yes, two tests
- ○ No
- ○ Unknown

Q8

Is this facility performing universal masking?

- Yes, of all healthcare personnel, visitors and patients
- Yes, of healthcare personnel and visitors
- Yes, of healthcare personnel only
- Yes, of visitors only
- No
- Unknown

Q9

Is this facility performing daily employee screening for signs and symptoms of COVID-19 at point of entry? (e.g., temperature checks, reported signs/symptoms, etc.)

- Yes
- No
- Unknown

Q10

Does this facility perform testing asymptomatic patients for COVID-19 before surgery?

- Yes
- No
- Unknown

Q11

What laboratory is this facility using to test for COVID-19?

- Quest
- Labcorp
- Viracor
- Abbott POC test
- Department of Health
- In House Testing
- Other: Enter lab name below

Q12

Does this facility perform antibody testing for COVID-19?

- Yes
- No
- Unknown

Q13

Any additional comments or more information related to questions above?

Add Block

End of Survey

Survey Termination Options...

Duke UNIVERSITY

Qualtrics.com Contact Information Legal
